# Overall survival and toxicity of Y90 radioembolization for hepatocellular carcinoma patients in Barcelona Clinic Liver Cancer stage C (BCLC-C)

**DOI:** 10.1186/s12876-022-02528-y

**Published:** 2022-11-17

**Authors:** Pulak Goswami, Oladapo R. Adeniran, Shelby K. Frantz, Lea Matsuoka, Liping Du, Ripal T. Gandhi, Zachary S. Collins, Marc R. Matrana, Michael Petroziello, Jayson S. Brower, Daniel Y. Sze, Andrew S. Kennedy, Jafar Golzarian, Eric A. Wang, Daniel B. Brown

**Affiliations:** 1 Vanderbilt University School of Medicine, Nashville, TN USA; 2 Division of Interventional Radiology, Vanderbilt University Medical Center, CCC-1118 Medical Center North, 1161 21st Ave S, Nashville, TN 37232 USA; 3 Division of Hepatobiliary Surgery and Liver Transplantation, Vanderbilt University Medical Center, Nashville, TN USA; 4 Department of Biostatistics, Vanderbilt University Medical Center, Nashville, TN USA; 5 Miami Cardiac and Vascular Institute/Miami Cancer Institute, Miami, FL USA; 6 Division of Interventional Radiology, University of Kansas, Kansas City, KS USA; 7 Division of Hematology/Oncology, Ochsner Medical Center, New Orleans, LA USA; 8 Division of Interventional Radiology, Roswell Park Medical Institute, Buffalo, NY USA; 9 Department of Radiology, Sacred Heart Medical Center, Spokane, WA USA; 10 Division of Interventional Radiology, Stanford University, Palo Alto, CA USA; 11 Department of Radiation Oncology, Sarah Cannon Research Institute, Nashville, TN USA; 12 Division of Interventional Radiology, University of Minnesota, Minneapolis, MN USA; 13 Department of Radiology, Carolinas Medical Center, Charlotte, NC USA

**Keywords:** Carcinoma, hepatocellular carcinoma, Yttrium radioisotopes/ therapeutic use, Yttrium radioisotopes/ adverse events, Adult, Treatment outcome

## Abstract

**Introduction:**

National Comprehensive Cancer Network HCC guidelines recommend Y90 to treat BCLC-C patients only in select cases given the development of systemic regimens. We sought to identify ideal candidates for Y90 by assessing survival and toxicities in this patient group.

**Materials and methods:**

The Radiation-Emitting Selective Internal radiation spheres in Non-resectable tumor registry is a prospective observational study (NCT: 02,685,631). Patients with advanced HCC were stratified into 3 groups based on tumor location, Eastern Cooperative Oncology Group (ECOG) performance status, and liver function. Group 1: liver isolated HCC, ECOG 0 and Child Pugh (CP) A (*n* = 12, 16%), Group 2: liver isolated HCC, ECOG ≥ 1 or CP B/C (*n* = 37, 49%), and Group 3: extrahepatic HCC with any ECOG or CP score (*n* = 26, 35%). Patients in any group could have macrovascular invasion. Overall survival (OS) and progression-free survival (PFS) with 95% confidence intervals (95% CI) were calculated. Grade 3 + toxicities were tracked using Common Terminology Criteria for Adverse Events v5. Cox proportional hazard model was performed to determine factors affecting OS.

**Results:**

Seventy-five BCLC-C patients treated between 2015 and 2019 were reviewed. The groups were similar in age, sex, race, and ethnicity (all *p* > 0.05). Bilobar disease was least common in Group 1 (*p* < 0.001). Median OS of the entire cohort was 13.6 (95% CI 7.5–16.1) months. Median OS of Groups 1–3 were 21.8, 13.1 and 11.5 months respectively (*p* = 0.6). Median PFS for the cohort was 6.3 (4.8–14.7) months. Median PFS for group 1 was not reached. Mean PFS for Group 1 was 17.3 ± 4.8 months. Median PFS for Groups 2 and 3 was 6.8 and 5.9 months (X^2^ = 1.5, *p* = 0.5). Twenty-four Grade 3 or greater toxicities developed, most commonly hyperbilirubinemia (8/75, 11%) and thrombocytopenia (2/75, 3%). The incidence of toxicities between groups was similar (all *p* > 0.05). Cox Proportional Hazard analysis predicted shorter OS with CP class B/C (X^2^ = 6.7, *p* = 0.01), while macrovascular invasion (X^2^ = 0.5, *p* = 0.5) and ECOG score of ≥ 1 (X^2^ = 2.1, *p* = 0.3) was not associated with OS.

**Conclusions:**

OS of CPA patients with advanced HCC and performance status of 0 was 21.8 months following Y90. CP A cirrhosis is the best predictor of prolonged OS in advanced (BCLC-C) HCC.

## Introduction

Hepatocellular carcinoma (HCC) is the sixth most diagnosed form of cancer and is the third leading cause of cancer death worldwide while continuing to increase in incidence [1–3]. Intra-arterial therapy with chemoembolization and trans-arterial radioembolization (TARE) are commonly used for patients with HCC [4, 5]. Survival by the Barcelona Clinic Liver Cancer classification (BCLC) decreases with advancing stage [6, 7]. Advanced/BCLC-C disease is defined by imaging criteria such as vascular invasion and/or extrahepatic disease in the setting of preserved liver function but also includes subjective criteria, including patients with an Eastern Cooperative Oncology Group (ECOG) performance status of 1–2. A criticism of including ECOG score is that patients may have symptoms related to underlying cirrhosis rather than from cancer and the ECOG score may not reflect cancer symptoms [8].

Several systemic therapy options including Atezolizumab-Bevacizumab, Nivolumab and Lenvatinib for patients with BCLC-C HCC have been developed in the last few years and are now accepted first-line therapy for advanced disease [4, 9–12]. Current National Cancer Cooperative Network recommendations state that patients with advanced HCC should be carefully evaluated prior to initiating locoregional therapy [4]. This statement reflects the relative lack of existing survival data when treating advanced HCC particularly with the development of efficacious systemic options [9]. These findings are compounded by the lack of success of Y90 to improve overall survival versus or in combination with sorafenib [13–15]. Findings in individual trials were reinforced by a metanalysis which found no benefit when adding Y90 to sorafenib [16]. Treatment of advanced HCC varies regionally. In the United States and Europe, locoregional therapy is primarily considered when systemic options fail or are poorly tolerated [17]. In other countries such as China, chemoembolization is recommended in the setting of advanced disease [18]. Previous evaluations of outcomes with TARE in BCLC-C patients reported on the use of glass microspheres [19]. Outcomes using resin microspheres have not been widely reported [20]. The Radiation-Emitting SIR-Spheres in Non-Resectable (RESiN) liver tumor registry (NCT 02,685,631) was a national multicenter, prospective observational study capturing data on demographics, laboratory parameters, treatment details, response and toxicities treated with resin microspheres. The registry captured real-world utilization of TARE outside the idealized scenario of clinical trials. The objectives of this study are to evaluate outcomes and toxicities from the registry in patients with BCLC-C HCC.

## Methods

### Registry/patients

The RESiN registry was an observational study collecting data on patients over 18 years of age with primary or secondary liver cancer scheduled to receive Y90 microsphere therapy as part of their treatment. The study was approved by the institutional review board at each of the 36 enrolling sites. The study protocol conformed to the ethical guidelines of the 1975 Declaration of Helsinki as reflected in a priori approval by each institution’s human research committee. All patients signed written informed consent. Physicians at each of the institutions determined appropriateness for treatment, Y90 dosimetry, and follow-up imaging and laboratory examination per local practice guidelines. Patients were enrolled on the day of treatment and tracked afterward with enrollment from 2015–2020. Key exclusion criteria included age less than 18 years of age, an inability to provide informed consent and previous TARE. Other prior hepatic interventions, such as resection, chemoembolization, ablation, and stereotactic body radiotherapy were allowed. Data were entered utilizing a Research Electronic Data Capture online database.

In this analysis, all patients had HCC diagnosed by radiologic appearance and/or biopsy. Of the 1655 patients enrolled in RESiN, 448 had HCC. Seventy-five patients were BCLC-C. The subgroups were determined by the presence or absence of portal vein invasion, extrahepatic metastatic disease, Child–Pugh (CP) class and ECOG performance status as suggested by Bolondi [21]. These categories were designed to identify patients who have biologically different forms of advanced disease and include patients who have:

1. Portal venous invasion and are ECOG 0 and CP A without extrahepatic disease.
2. Portal venous invasion and are ECOG 1–2 and/or CP B-C without extrahepatic disease
3. Have extrahepatic disease with or without portal venous invasion and any ECOG CP score.

### Procedures

Participants were treated by trained interventional radiologists to ensure minimal patient and operator radiation exposure [22]. Patients underwent mapping scintigraphy with technetium 99 m-labeled macroaggregated albumin to ensure lung dose < 30 Gray as well as absence of extrahepatic deposition. Based on these findings, therapeutic dose was calculated and patients then underwent TARE with resin ^90^Y microspheres. The procedures were performed in accordance to the quality improvement guidelines of the Society of Interventional Radiology [23].

Imaging and Response Assessment:

Baseline and follow-up imaging consisted of multiphase contrast-enhanced CT or MRI scans. The tumor number, location and sizes were calculated along with the total tumor diameter in patients with measurable disease. Total tumor diameter was defined as the diameter of a single tumor or the sum of the maximal diameters in the setting of multifocal disease. Measurable disease was defined as tumors where margins could be accurately assessed to calculate greatest diameter. Portal vein patency and/or level of invasion was assessed as well. Timing of follow-up imaging was per institutional guidelines with response determined using modified Response Evaluation Criteria in Solid Tumors (mRECIST) criteria. Studies were assessed by trained diagnostic radiologists to limit inter-operator variability described in other studies [24].

### Data analysis

The Kruskal–Wallis test was used to calculate continuous variables and the Pearson test was used for discrete variables. Overall (OS) and Progression-free survival (PFS) were defined as the time from the date of treatment to death or confirmation of disease progression at any site, respectively. Kaplan–Meier analysis was performed to compare OS and PFS with 95% confidence intervals reported. Data regarding the incidence of adverse events (AEs) was tracked using the Common Terminology Criteria for Adverse Events version 5 with grades 1–5. If a patient had multiple events of the same AE within the course of the study, then the highest grade was given and counted as a single event. A single patient could develop multiple AEs. A Cox Proportional Hazards model was performed to identify which baseline factors predicted longer OS.

## Results

### Demographics

The cohort and subgroup details are outlined in Table 1. The majority of patients were male (*n* = 56, 75%), white (*n* = 56, 75%), and non-Hispanic (*n* = 65, 87%). Groups 1–3, had 12, 37, and 26 patients, respectively. The subgroups were similar in age (*p* = 0.921), gender (*p* = 0.939), race (*p* = 0.735), and ethnicity (*p* = 0.499). The baseline serum bilirubin was significantly higher in Group 2 compared to Groups 1 and 3 (median = 1.1 mg/dl versus 0.8 and 0.7 mg/dl, respectively, *p* = 0.023) as was aspartate transaminase (median = 68.5 U/L versus 37.0 and 44.5 U/L respectively, *p* = 0.039). The cause of cirrhosis was similar across all groups (*p* = 0.87) with hepatitis C the most common etiology. Non-alcoholic steatohepatitis was more common in Group 1 (4/12, 33%) than in Groups 2 (3/26, 12%) or 3 (1/37, 3%) (*p* = 0.01). The median Model for End-Stage Liver Disease (MELD) score of Group 2 [10, interquartile range (IQR 8–12)] was significantly higher (*p* < 0.001) than Groups 1 (median 8, IQR 7–9.5) and 3 (median 7, IQR 6–9). The percentage of patients with Child B/C cirrhosis was significantly higher (*p* < 0.001) in Group 2 (22/36, 61%) than Groups 1 (0/12, 0%) and 3 (4/22, 18%).

**Table 1:** Patient Demographics for group 1, 2, and 3

	*N* reported	Group 1 (*N* = 12)	Group 2 (*N* = 37)	Group 3 (*N* = 26)	Combined	*P*-value
*Age*	74	63.0 (58.2–68.2)	64.0 (58.0–68.0)	63.0 (59.0–69.0)	63.0 (58.2–69.0)	0.921
*Gender*	75					0.939
Female		3 (25%)	10 (27%)	6 (23%)	19 (25%)	
Male		9 (75%)	27 (73%)	20 (77%)	56 (75%)	
*Race*	75					0.735
American Indian /Alaska		0 (0%)	1 (3%)	0 (0%)	1 (1%)	
Asian		0 (0%)	2 (5%)	1 (4%)	3 (4%)	
Black		0 (0%)	4 (11%)	4 (15%)	8 (11%)	
Native Hawaiian		0 (0%)	1 (3%)	0 (0%)	1 (1%)	
Other		0 (0%)	1 (3%)	1 (4%)	2 (3%)	
Unknown		2 (17%)	1 (3%)	1 (4%)	4 (5%)	
White		10 (83%)	27 (73%)	19 (73%)	56 (75%)	
*Ethnicity*	75					0.499
Hispanic or Latino		1 (8%)	1 (3%)	2 (8%)	4 (5%)	
Non-Hispanic		9 (75%)	34 (92%)	22 (85%)	65 (87%)	
Unknown		2 (17%)	2 (5%)	1 (4%)	5 (7%)	
Other		0 (0%)	0 (0%)	1 (4%)	1 (1%)	
*Causes of Cirrhosis*						
Alcohol	75					0.094
Yes		2 (17%)	11 (30%)	2 (8%)	15 (20%)	
No		10 (83%)	26 (70%)	24 (92%)	60 (80%)	
Hepatitis B	75					0.508
Yes		0 (0%)	3 (8%)	1 (4%)	4 (5%)	
No		12 (100%)	34 (92%)	25 (96%)	71 (95%)	
Hepatitis C	75					0.869
Yes		5 (42%)	15 (41%)	9 (35%)	29 (39%)	
No		7 (58%)	22 (59%)	17 (65%)	46 (61%)	
NASH	75					0.011
Yes		4 (33%)	1 (3%)	3 (12%)	8 (11%)	
No		8 (67%)	36 (97%)	23 (88%)	67 (89%)	
Other	75					0.908
Yes		1 (8%)	2 (5%)	2 (8%)	5 (7%)	
No		11 (92%)	36 (97%)	23 (88%)	67 (89%)	
*Bilirubin*	74	0.800 (0.675–0.925)	1.05 (0.600–1.625)	0.700 (0.500–0.900)	0.800 (0.600–1.300)	0.023
*Albumin*	74	3.60 (3.18–3.92)	3.50 (3.20–3.68)	3.35 (3.08–3.60)	3.50 (3.10–3.70)	0.324
*Ascites*	75					0.014
Yes		2 (17%)	16 (43%)	3 (12%)	21 (28%)	
No		10 (83%)	21 (57%)	23 (88%)	54 (72%)	
*Hepatic Encephalopathy*	75					0.333
Yes		1 (8%)	1 (3%)	0 (0%)	2 (3%)	
No		11 (92%)	36 (97%)	26 (100%)	73 (97%)	
*MELD*	70	8.0 (7.0–9.5)	10.0 (8.0–12.0)	7.0 (6.0–9.0)	9.0 (7.0–11.0)	< 0.001
*Child Pugh Class*	70					< 0.001
Class A		12 (100%)	14 (39%)	18 (82%)	44 (63%)	
Class B/C		0 (0%)	22 (61%)	4 (18%)	26 (37%)	

### Imaging

Baseline CT and MR findings are outlined in Table 2. Bilobar disease was most common in Group 2 (16/26, 62%) compared to Groups 1 (1/12, 8%) and 3 (8/37, 22%) (*p* < 0.001). Both the number (*p* = 0.09) and tumor diameter (*p* = 0.06) were similar between groups. Thirty-eight patients (51%) had multifocal disease. Total tumor diameter was assessable in 49 patients (65%) with indistinct tumor margins limiting assessment in the remaining participants. Tumor thrombus was significantly more common in Groups 1 (12/12, 100%) and 2 (37/37, 100%) compared to Group 3 (6/20, 30%, *p* < 0.001). Ascites was more common in Group 2 (*n* = 16/37, 43%), than in Group 1 and Group 3 (*n* = 2/12, 17% and *n* = 3/26, 12% respectively, *p* = 0.014).

**Table 2:** Baseline imaging findings

	*N* reported	Group 1 (*N* = 12)	Group 2 (*N* = 37)	Group 3 (*N* = 26)	Combined	*p*-value
*Tumor Number*	73					0.09
1		7	21	7	35 (48%)	
2–3		1	8	7	16 (21%)	
> 4		4	6	12	22 (30%)	
*Tumor Location*	75					< 0.001
Bilobar		1	8	16	25 (33%)	
One Lobe		11	29	10	50 (67%)	
*Total Tumor Diameter (cm)*	49	6.4 (3.2–13.6)	12.9 (7.5–16.1)	15.1 (10.6–22.3)	12.9 (7.0–20.0)	0.06
*Vascular Invasion*	69	12	37	6	55 (80%)	< 0.001
Patent		0 (0%)	0 (0%)	14 (70%)	14 (20%)	
Segmental Thrombosis		5 (42%)	11 (30%)	2 (10%)	18 (26%)	
Lobar Thrombosis		2 (17%)	12 (32%)	3 (15%)	17 (25%)	
Main Thrombosis		5 (42%)	14 (38%)	1 (5%)	20 (29%)	

Total tumor diameter refers to the diameter of a single tumor or the sum of the maximal measurable diameters in the setting of multifocal disease

### Dosimetry

Dosimetry methodology was available in 51/75 (68%) patients: 11/12 (92%) in Group 1, 15/26 (58%) in Group 2, and 25/37 (68%) in Group 3. The most common method was body surface area method in 10/12 (83%) in Group 1, 13/15 (87%) in Group 2 and 23/25 (92%) in Group 3. The difference in dosimetry method was not significant by group (p = 0.5). Median prescribed activity between groups was also not significantly different (*p* = 0.7): 1.3 GBq (IQR: 1.2–1.5) in Group 1, 1.5 GBq (IQR: 1.1–1.8) in Group 2, and 1.5 (1.0–1.8) in Group 3. Treatment location was also similar between groups with lobar infusions most common: 10/12 (83%) in Group 1, 20/26 (77%) in Group 2 and 25/37 (68%) in Group 3 (*p* = 0.3). No repeat therapies were reported.

### Survival

**Fig. 1:**
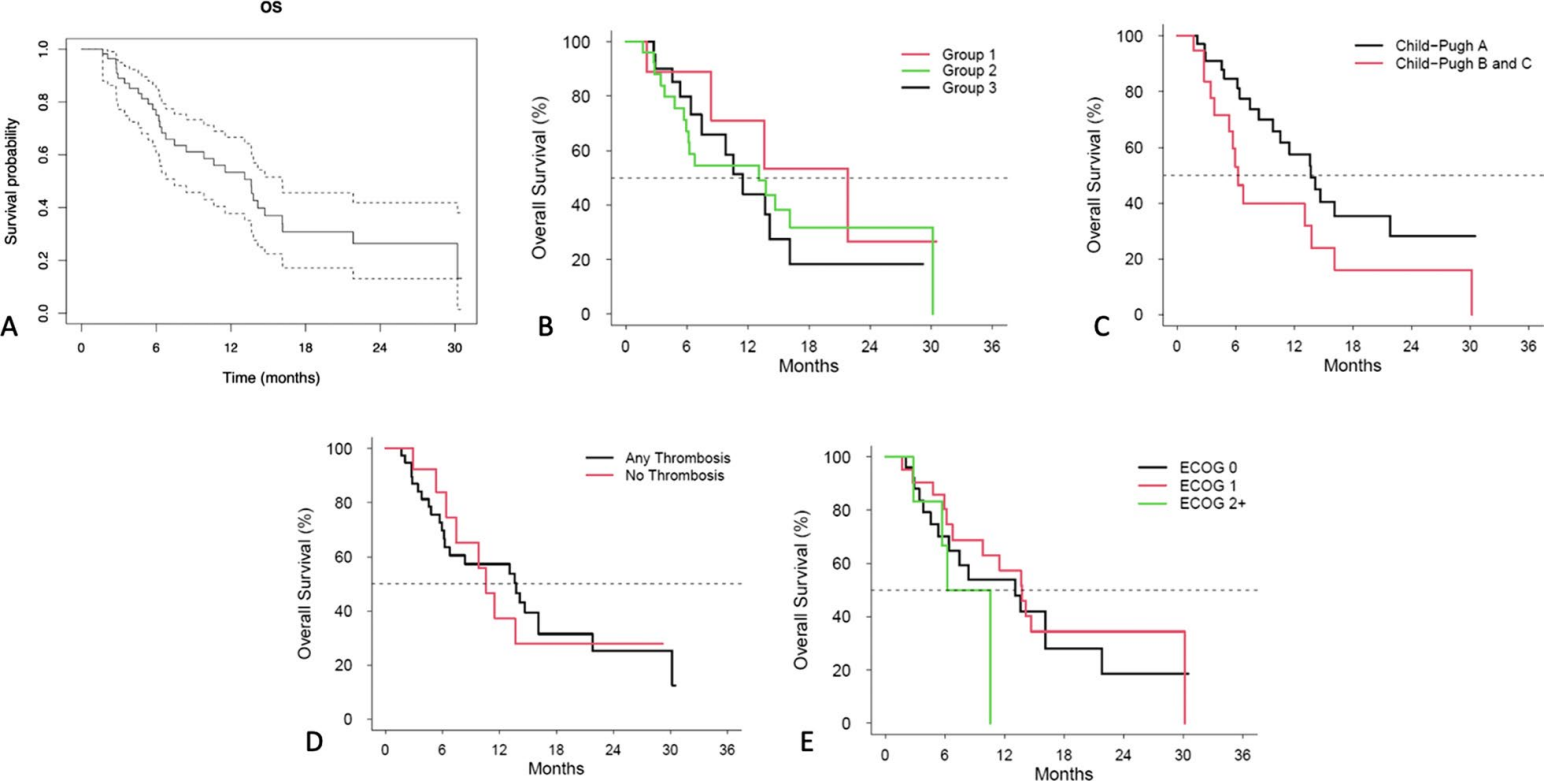
Overall survival of (**A**) the entire cohort: 13.6 months (95% CI: 7.5–16.1 months), (**B**) the 3 subgroups (**C**) child Pugh A versus Child Pugh B/C patients (**D**) with versus without portal vein thrombosis (**E**) with Eastern Cooperative Oncology Group performance scores of 0 versus 1 versus 2 or greater

Median OS of the entire cohort was 13.6 (95% CI 7.5–16.1) months (Fig. 1A). There were no deaths within 30 days of treatment. Median OS of Groups 1–3 were 21.8 (95% CI 2.1—Not Reached), 13.1(15% CI 5.7- Not Reached) and 11.5 (95% CI: 6.4—16.1) months respectively (Fig. 1B). These differences were not statistically significant (X^2^ = 0.9, *p* = 0.6). The median OS for CP class A and CP class B/C were 13.67 (95% CI 8.4—21.8) and 6.28 (3.8—13.8) respectively (X^2^ = 3.8, *p* = 0.05) (Fig. 1C). The median OS with venous invasion (Fig. 1D) was 13.8 months (95% CI 6.2–16.2) and not significantly different than with a patent portal venous system with a median of 10.6 months (95% CI: 5.4—Not Reached, X^2^ = 0, *p* = 1). Similarly, a performance status (Fig. 1E) of 0 (median 13.1 months, 95% CI: 5.4–21.8), 1 (median 13.8 months, 95% CI: 6.2—Not Reached) and 2 (median 8.4 months, 95% CI: 2.8—Not Reached, X^2^ = 1.5, *p* = 0.5) did not affect OS.

**Fig. 2:**
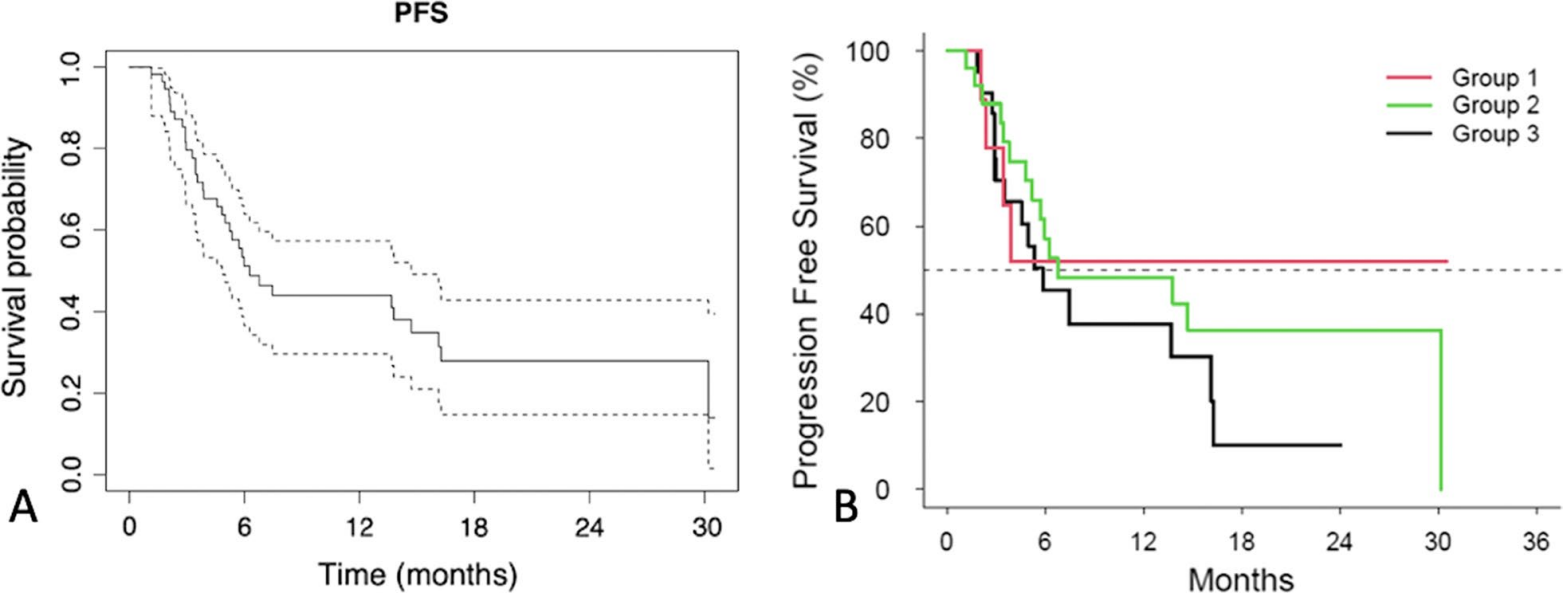
Progression-free survival of (**A**) the entire cohort (**B**) the 3 subgroups

Median PFS for the cohort was 6.3 (95% CI: 4.8–14.7) months (Fig. 2A). Median PFS for Group 1 was not reached at 17.3 months mean. PFS for Groups 2 and 3 was 6.8 (95% CI 4.83—Not Reached) and 5.9 (2.96, 16.1) months (X^2^ = 1.5, *p* = 0.5) (Fig. 2B).

### Response/progression

Six-month imaging was performed in 38 patients (51%) with 35 patients (47%) having response assessment. Fourteen percent had complete response (5/35), 17% had a partial response (6/35), 37% had stable disease (13/35), and 31% had progressive disease (11/35). The objective response rate was 31% (11/35 patients) and the disease control rate was 68% (24/35 patients).

Details of progressive disease were available in 25 patients (33.3%) from the entire cohort. All 25 patients developed intrahepatic progression and 12% (3/25) also developed extrahepatic disease. Regarding intrahepatic progression, 28% (7/25) developed progression outside and 72% (18/25) developed progression within the treated region. The incidence of progressive disease was similar between the groups (all *p* > 0.05).

### Off-study

Fifty (67%) of the 75 patients left the study. Seventy eight percent (39/50) died, 12% (6/50) were lost to follow up, and 10% (5/50) entered hospice. The cause of death was available for 24 of the 39 (62%) who expired. Eighty four percent (20/24) died from tumor progression or worsening cirrhosis. The remaining 17% (4/24) died of other causes.

### Toxicity

Twenty-seven Grade 3 or greater toxicities developed and are outlined in Table 3. There were 16 Grade 3 or greater hepatic function toxicities in 12 patients with hyperbilirubinemia (8/58, 14%) and elevated alanine aminotransferase (3/58, 5%) being the most common. There were no liver function adverse events within 30 days. Thirteen of the 16 events developed in patients with progressive disease, leaving only 3 hepatic function toxicities that were directly attributable to the procedure: 1 (2%) Grade 3 hyperbilirubinemia and 2 (3%) alanine transaminase elevations. Four percent of patients developed thrombocytopenia (3/75). The incidence of toxicities between groups was similar (all *p* > 0.05). There was one Grade 5 event which was reported as a death with no additional information.

**Table 3:** Summary of grade 3–4 toxicities

Toxicity	Grade 3	Grade 4	Total
*Liver Function Adverse Events*			
Bilirubin (*N* = 58)	7	1	8
Albumin (*N* = 58)	1	0	1
AST (*N* = 58)	1	1	2
ALT (*N* = 58)	2	1	3
INR (*N* = 48)	0	0	0
*Other Laboratory Adverse Events*			
Thrombocytopenia	3	0	3
Leukopenia	1	0	1
*Constitutional Adverse events*			
Fever	1	0	1
Abdominal Pain	1	0	1
Flank Pain	1	0	1
Abdominal Distention	1	0	1
Nausea	1	0	1
Non-cardiac chest pain	1	0	1
Stomach Pain	1	0	1
Tumor lysis Syndrome	1	0	1
Urinary tract infection	1	0	1
Total:	24	3	27

*AST* aspartate aminotransferase, *ALT* alanine aminotransferase, *INR* international normalized ratio

### Cox proportional hazards model

The full Cox Proportional Hazard model is shown in Table 4. Only one factor, CP class A versus CP class B/C predicted shorter OS (X^2^ = 6.7, *p* = 0.01), whereas macrovascular invasion (X^2^ = 0.5, *p* = 0.5) and ECOG score of ≥ 1 (X^2^ = 2.1, *p* = 0.3) were not associated with OS.

**Table 4:** Cox Proportional Hazard regression of baseline risk factors predicting survival events

Value	Coefficient	Z	Hazard ratio	*p*-value
Child–Pugh B/C	1	0.4	2.6	0.009
ECOG 1	− 0.1	0.4	− 0.3	0.8
ECOG 2 +	0.5	0.4	1.2	0.2
Portal Vein Invasion	0.3	0.4	0.7	0.5

Child–Pugh B or C status was associated with survival events

## Discussion

The current work demonstrates that treatment of BCLC-C patients with resin Y90 microspheres is safe and effective. The patients most likely to benefit from resin Y90 microsphere therapy were patients who were ECOG 0 and CP class A with disease confined to the liver. This group lived a median of 21.8 months. CP class A cirrhosis was the primary predictor of longer OS in a Proportionate Hazard model that also included ECOG and venous invasion. No deaths occurred within 30 days and the toxicity profile was not severe with attributable Grade 3 or greater hepatic function toxicities in 5% (3/58) of patients and other toxicities in 14% (8/58) of patients. TARE should be considered in patients who are intolerant of or who progress on systemic therapy.

The 21.8-month OS of ECOG 0, CP A patients with advanced HCC in the current trial is similar to Atezolizumab/Bevacizumab from the Imbrave150 trial (9). The drug combination is currently the recommended first-line treatment for CP A patients with advanced HCC with a median OS of 19.2 months (2;9). The study group in Imbrave150 were all CP A and included 18% with BCLC A or B disease compared to 45% CP A and 0% BCLC A or B in the current study. The PFS in the current cohort (7.5 months) was similar to the Imbrave150 study group (6.9 months). The current results also compare favorably to Lenvatinib and Nivolumab (7;8). The Lenvatinib and Nivolumab trials included 18% and 21.8% BCLC A and B patients and also only enrolled CP A patients. This approach resulted in median OS of 13.6 and 16.4 months for Lenvatinib and Nivolumab, respectively, compared to the whole group OS of 13.7 months in the current study. The rate of tumor vascular invasion in the current trial (81%) was higher than Imbrave150 (38%), Lenvatinib (20.9%) and Nivolumab (23%). Despite these baseline differences, the Grade 3 or greater toxicity rates were no greater with radioembolization than with the current standard of care therapies for advanced HCC. Differences between these trials are outlined in Table 5.

**Table 5:** Summary of the differences in OS, PFS, toxicities, number of BCLC A or B patients, and number of patients without vascular invasion or extrahepatic metastasis

	Current study	Atezolizumab –bevacizumab	Nivolumab	Lenvatinib
Number of BCLC A or B patients (proportion)	0 (0%)	60 (18%)	68 (18%)	104 (21.8%)
Number of patients with no vascular invasion (proportion)	13 (19%)	91 (62%)	247 (77%)	755 (79.1%)
Child Pugh A	34 (45%)	336 (100%)	371 (100%)	478 (100%)
Overall Survival in months (95% CI)	13.7 (8.41–21.8)	19.2 (17.0 – 23.7)	16.4 (13.9–18.4)	13.6 (12.1–14.9)
Progression free survival in months (95% CI)	7.46 (3.55–16.26)	6.9 (5.7–8.6)	3.7 (3.1–3.9)	7.4 (6.9–8.8)
≥3 Toxicities	12 (16%)	143 (43%)	81 (22%)	270 (56.7%)

The current cohort also is comparable to a previous evaluation of radioembolization using glass microspheres in patients with advanced HCC [8]. Ali, et al. reported whole group OS of 10.7 months. Their group included 202 patients (36.9%) with multiple reasons for BCLC-C status and also 345 patients (63.1%) with a single source for BCLC-C diagnosis. The single etiology group was separated into 233 patients with ECOG score related BCLC-C and 112 patients with vascular invasion or extrahepatic disease. They described a longer median OS (12.9 months) with an ECOG score of 1 compared to 0 (8.7 months) and 2 (4.3 months). Group 2 in the current study included 37 patients who had ECOG scores of 1–2. All 37 patients had vascular invasion as well. The absence of single component causes in the current study group makes it difficult to directly compare the current results to the results from Ali, et al. [8]. Choi, et al. reported use of chemoembolization alone or combined with chemoinfusion in patients with advanced HCC and reported a median OS of 15.5 months [25]. They noted improved outcomes noted when chemoinfusion was added to chemoembolization.

The incidence of grade ≥ 3 AE in our study were comparable to treatment with Nivolumab (*n* = 82/367, 22%) and less than Lenvatinib (*n* = 270/476, 56.7%), and Atezolizumab/Bevacizumab (*n* = 207/329, 63%) [10–12]. Concerns about toxicity with radioembolization for advanced HCC should not preclude therapy when compared to the other treatment options.

HCC screening prior to diagnosis was not tracked as part of the current study. The current HCC screening recommendations in the United States include ultrasound imaging with alpha-feto protein measurement [26]. Even in the setting of optimal surveillance utilization, some HCC’s are sonographically undetectable, particularly in patients with non-alcoholic steatohepatitis (11% of the current group) [27]. Radioembolization has longer OS when diagnosed at earlier stages: Frantz, et al. did not reach median OS at 30 months for BCLC A patients and reported a median OS of 19.5 months in BCLC B patients [7]. As screening with MRI undergoes further evaluation, a higher rate of early detection may be possible employing either non-contrast or liver-contrast specific MRI [28, 29]. Additionally, as dosimetry methods for Y90 evolve with use of multicompartment partition dosing, survival may increase further. This outcome is currently being investigated in an ongoing prospective trial [30].

Our study is limited as a single arm cohort which has a modest sample size, a factor that is most apparent in the subgroup analyses. A larger sample size may have identified other differences between the three subgroups. Additionally, our analysis of PFS is challenging due to the difficulty in assessing response to Y90 in advanced HCC due to the incidence of amorphous tumor boundaries and challenges in assessing changes with vascular invasion. This challenge may be a reason PFS wasn’t reported in other radioembolization studies [8]. There was also less than 100% data entry. Despite these limitations, we were able to report OS and toxicity rates that are similar to other therapies recommended by NCCN.

## Conclusion

The current study found that BCLC-C patients treated with resin Y90 had OS comparable to those identified with Lenvatinib and Nivolumab. Additionally, patients with CP A cirrhosis and performance status of 0 had OS of almost 22 months, similar to the outcomes of Atezolizumab and Bevacizumab. These findings were achieved with a reasonable toxicity profile. TARE with resin microspheres remains a reasonable option for patients with advanced HCC.

## Data Availability

All data generated or analyzed during this study are included in this article. Further enquiries can be directed to the corresponding author.

## Abbreviations

AE: Adverse events
BCLC: Barcelona clinic liver cancer stage
ECOG: Eastern cooperative oncology group
HCC: Hepatocellular carcinoma
IQR: Interquartile range
MELD: Model for end-stage liver disease
OS: Overall survival
PFS: Progression-free survival
RESiN: Radiation-emitting SIR-spheres in non-resectable liver tumor
TARE: Trans-arterial radioembolization
